# Pan-cancer analysis identifies the correlations of Thymosin Beta 10 with predicting prognosis and immunotherapy response

**DOI:** 10.3389/fimmu.2023.1170539

**Published:** 2023-05-18

**Authors:** Zhanzhan Li, Yanyan Li, Yifu Tian, Na Li, Liangfang Shen, Yajie Zhao

**Affiliations:** ^1^ Department of Oncology, Xiangya Hospital, Central South University, Changsha, Hunan, China; ^2^ Department of Nursing, Xiangya Hospital, Central South University, Changsha, Hunan, China; ^3^ Department of Pathology, Xiangya Hospital, Central South University, Changsha, Hunan, China; ^4^ Department of Nuclear Medicine, Xiangya Hospital, Central South University, Changsha, Hunan, China; ^5^ National Clinical Research Center for Geriatric Disorders, Xiangya Hospital, Central South University, Changsha, Hunan, China

**Keywords:** TMSB10, pan-cancer, immunotherapy, prognosis, immune infiltration

## Abstract

**Introduction:**

The biological function and prognosis roles of thymosin β(TMSB) 10 are still unclear in pan-cancer.

**Methods:**

We retrieved The Cancer Genome Atlas and Genotype-tissue expression datasets to obtain the difference of TMSB10 expression between pan-cancer and normal tissues, and analyzed the biological function and prognosis role of TMSB10 in pan-cancer by using cBioPortal Webtool.

**Results:**

The expression of TMSB10 in tumor tissues was significantly higher than normal tissues, and showed the potential ability to predict the prognosis of patients in Pan-cancer. It was found that TMSB10 was significantly correlated with tumor microenvironment, immune cell infiltration and immune regulatory factor expression. TMSB10 is involved in the regulation of cellular signal transduction pathways in a variety of tumors, thereby mediating the occurrence of tumor cell invasion and metastasis. Finally, TMSB10 can not only effectively predict the anti-PD-L1 treatment response of cancer patients, but also be used as an important indicator to evaluate the sensitivity of chemotherapy. In vitro, low expression of TMSB10 inhibited clonogenic formation ability, invasion, and migration in glioma cells. Furthermore, TMSB10 may involve glioma immune regulation progression by promoting PD-L1 expression levels via activating STAT3 signaling pathway.

**Conclusions:**

Our results show that TMSB10 is abnormally expressed in tumor tissues, which may be related to the infiltration of immune cells in the tumor microenvironment. Clinically, TMSB10 is not only an effective prognostic factor for predicting the clinical treatment outcome of cancer patients, but also a promising biomarker for predicting the effect of tumor immune checkpoint inhibitors (ICIs) and chemotherapy in some cancers.

## Introduction

The thymosin β(TMSB)family members, including TMSB4, TMSB10 and TMSB15, which were originally identified from the thymus. The functions of TMSB mainly to inhibit actin polymerization and disrupt F-actin formation. TMSB10 contains 40-44 amino acid protein and is mainly localized in cytoplasm, which has multiple physiological functions in humans, such as early organ development, apoptosis, proliferation, migration, angiogenesis (1).

Several studies have found that up-regulated expression of TMSB10 is associated with metastasis and invasion in a variety of solid cancers. For example, Overexpression of TMSB10 by activating the AKT/FOXO signaling pathway *in vitro* and *in vivo* could promote proliferation, invasion, and migration of breast cancer (2–4). By mediating the transformation and proliferation of pI3k/AKT signaling pathway, TMSB10 could promote lung adenocarcinoma (2–4). TMSB10 induces renal cell carcinoma by regulating renal epithelial mesenchymal transition (2–4). It is also a key factor in promoting the proliferation of papillary thyroid carcinoma (PTC) and epithelial-mesenchymal transition (EMT) progression, by negatively regulating microRNA (5). Recently, several reports have found that TMSB10 may have a closely relationship with immune infiltration, JUN as one of the activating protein-1 (AP-1) transcription factor, it regulates the expression of TMSB10 through transcription by CHIP assay, which could enrich its biological information function (6, 7).

In our study, we conducted a pan-cancer genomic analysis of TMSB10 across different cancer types by using GTEx and The Cancer Genome Atlas (TCGA) database, evaluating the expression of TMSB10 and its association with the prognosis of patients with different cancers. Furthermore, we examined the relationship between TMSB10 expression and the immune cell infiltration score, immune checkpoints, immune activation genes, immune inhibition genes and the response of immunotherapy and chemotherapy. Finally, we validated our findings *in vitro*. Our research aims to provide a new understanding of TMSB10 in Pan-cancer. The results show that TMSB10 has the potential to affect the tumor microenvironment, cancer immunotherapy and chemotherapy response.

## Methods

### Data collection and processing

TMSB10 expression in different tissues is based on The Cancer Genome Atlas (TCGA) pan-cancer tissue database and Chinese Glioma Genome Atlas (CGGA), normal human tissue data is based on Genotype-Tissue Expression (GTEx) database, which were downloaded from the UCSC Xena database (https://xenabrowser.net/datapages/) (8). The marked copy number segment, DNA methylation (Illumina human methylation 450), gene expression RNAseq (HTSeq), somatic mutation (SNPx and small INDELs) were also downloaded. The expression profile was converted into transcripts in the format of millions of bases per thousand (TPM) according to the following steps: We call raw data as “read counts”, “total reads” are sum of read counts of all genes in each sample. We can obtain a matrix data including genes through the read counts divided by the length of the gene. Finally, we obtained the relative expression data matrix data divided by “total reads”, and the data in log2 (TPM+1) format were used for subsequent analysis. All cancer lists with abbreviations was in the **Table S1**.

### Genomic alterations, localization, and interaction of TMSB10 in cancers

Using multifunctional cBioPortal cancer genome database (http://www.cbioportal.org) can identify molecular data in cancer tissue and understand related gene epigenetics, gene expression and protein group (9, 10). In this study, we explored the correlations of TMSB10 mRNA expression and coy number variation in cancer through this database, such as gene alteration frequency, gene mutation, gene amplification and deep deletion. We also visualized the rate of change in the genome through the cBioPortal Webtool.

The Human Protein Atlas (HPA; http://www.proteinatlas.org) database was used to provide the protein level of TMSB10 in human tumor. String (https://string-db.org/) database was used to show the protein-protein interaction network (PPI; http://comppi.linkgroup.hu/) of TMSB10. GeneCards (https://www.genecards.org/) was used to visualize the subcellular locations of TMSB10.

### Prognostic and function enrichment analysis

Prognostic factors included overall survival (OS) time, progression-free survival (PFS) time, disease-specific survival (DSS) time, and disease-free interval survival (DFI) time. Kaplan-Meier model and Univariate Cox Regression were used to evaluate the relationship between TMSB10 and pan-cancer.

50 Hallmark gene sets were obtained from the Molecular Signature Database (MSigDB, https://www.gseamsigdb.org/gsea/index.jsp) and the Normalized Enrichment Fraction (NES) and False Discovery Rate (FDR) of biological processes of each cancer were calculated. The R software packages “clusterProfiler” (11) and “GSVA” (12) were used for gene enrichment analysis, and the results were displayed as heat maps in the R software package “ggplot2”.

### Immune infiltration of TMSB10

Tumor microenvironment (TME) plays an important role in tumor genesis and development. By ImmuneScore, StromalScore and ESTIMATE Score, we found that the higher the ImmuneScore or StromalScore was, the larger the proportion of immune matrix was, which was positively correlated with immune infiltration. ESTIMATE Score is the sum of the Immune Score and Stromal Score, which represents the time of the integral proportional component of ESTIMATE Score. We evaluated the relationship between TMSB10 mRNA expression and several immune cell subsets, including cancer-associated fibroblast (CAF), B cells, neutrophils, CD4+ T cells, endothelial cells (Endo), eosinophil (Eos), NK T cells, γ/δ T cells, monocytes, macrophages, CD8+ T cells, mast cells, and NK cells across cancers in a heatmap by using the R package “ggplot2”.

### Immunotherapy prediction

The Spearman correlation analysis was used to analyze the association between TMSB10 and immunotherapy biomarkers. This analysis could also reflect the relationship between TMSB10 and tumor mutation load (TMB) and satellite instability (MSI) in pan-cancer. In order to explore the relationship between TMSB10 and immune checkpoint blockade (ICB), two ICB therapy cohorts, anti-PDL1 (CD274) and CTLA4, were used to verify the ability of TMSB10 in immunotherapy response in renal carcinoma and melanoma. By using the “SURV cut point” of the “SurvMiner” R software package to determine the optimal cutoff value, patients were divided into low-expression TMSB10 group and high-expression TMSB10 group. Chi-square test was used to assess the Overall Survival and Progression Free Survival in the patients with low and high TMSB10 expression.

### Chemotherapy sensitivity

The Spearman method was used to analyze the relationship between TMSB10 and chemotherapy sensitivity. IC50, the semi-inhibitory concentration, measures the concentration of 50% of tumor cell apoptosis induced by chemotherapy drugs, it can reflect the tolerance degree of tumor cells to various chemotherapy drugs. The higher of the IC50, the stronger resistant ability of the tumor. IC50 was used to investigate the relationship between TMSB10 and chemotherapy sensitivity. The Connectivity Map (CMap) database (https://portals.broadinstitute.org/cmap/), which could provide the relationship between TMSB10 expression in pan-cancer and specific inhibitors through the heatmap.

### Cell lines and western blotting

Human astrocytes were purchased from BIONEED (Beijing, China), and U251 and LN229 were purchased from the BeNa Culture Collection (Beijing). All cells were cultured in DMEM (Sigma-Aldrich, USA) with 10% fetal bovine serum (FBS) at 37 °C with 5% CO_2_. TMSB10 and negative control (NC) were synthesized by Santa Cruz Biotechnology (USA). Cell transfection was done using 40 nM *TMSB10* RNA or vector for 24 hours. The cell proteins were extracted using RIPA buffer. Protein concentrations were detected using a BCA kit. Incubation of the membranes was done with rabbit anti-human primary antibodies against TMSB10 (Cell Signaling Technology, USA), E-cadherin, N-cadherin, and Vimentin (Cell Signaling Technology) or β-tubulin (Cell Signaling Technology) as a loading control.

### Reverse-transcription quantitative polymerase chain reaction

RT-qPCR was performed as per the guidelines provided by the manufacturer (Takara Bio, Japan). TRIzol kit (Invitrogen, USA) was utilized for total RNA extraction, and cDNA was formed by reverse transcription of 1,000ng RNA in 20μL reaction volume. Santa-Cruz Biotechnology designed the following qPCR primer sequences: reverse 3`- cttatcgaagctggcgattt -5` and forward 5`- agtgggagcaccaggatct -3`. SYBR premix Taq and a CFX96 Real-Time PCR Detection System (Bio-Rad, USA) were employed to perform RT-qPCR. For the relative expression quantification of TMSB10, the 2^-ΔΔCT^ method was utilized.

### Cell migration assay

24-well transwell chambers were utilized to conduct cell migration assay with 600μL DMEM and 10% FBS at the bottom. NC and glioma (U251 and LN229) cells (1 × 10^5^) with transfection of TMSB10 were seeded into upper chambers with 100µL serum-free medium. Cotton swabs were utilized to remove cells on the upper surface of the filter following a whole day of culture. In addition, 4% formaldehyde was utilized to fix lower-surface invading cells, followed by their staining using Giemsa solution.

### Cell scratchy assay

Horizontal lines were drawn across the back of a six-well plate using a marker at approximately 0.5–1 cm distance from each other. Approximately 1 x 10^5^ NC shRNA and U251 and LN229 TMSB10-overexpressing cells were seeded into the six-well plate. A sterile 200-μL pipette tip was employed to scrape the cell monolayer after two days. This was followed by cell washing thrice using PBS to remove loose cells and adding the serum-free medium, after which cell incubation was done at 37°C with 5% CO_2_. Photographs were recorded at 0 and 48 hours.

### Clonogenic assay

NCs and U251 and LN229 TMSB10-overexpressing cells were seeded in six-well plates (1 × 10^5^ cells per well). PBS was employed to wash the cells thrice after a period of 14 days, and then they were stained using 0.2% crystal violet. The surviving colonies were identified as those with a cell number greater than 50. Viability data were standardized based on the NC treatment.

### Statistical analyses

The Wilcoxon rank-sum test was used to compare the expression difference of TMSB10 between tumor tissues and normal tissues. Paired T test was used to evaluate the protein levels of TMSB10 in clinical GBM samples and adjacent tissues. Univariate Cox regression analysis and Kaplan-Meier method (log-rank test) were used to evaluate the effect of TMSB10 expression on the prognosis of generalized carcinoma. The Spearman correlation analysis was used to predict the efficacy of TMSB10 on immunotherapy checkpoint inhibitors and chemotherapy. Finally, chi-square test was used to compare low and high expression of TMSB10 with specific inhibitors in pan-cancer.

## Results

### Abnormal expressions of TMSB10 in pan-cancer

**Figure 1:**
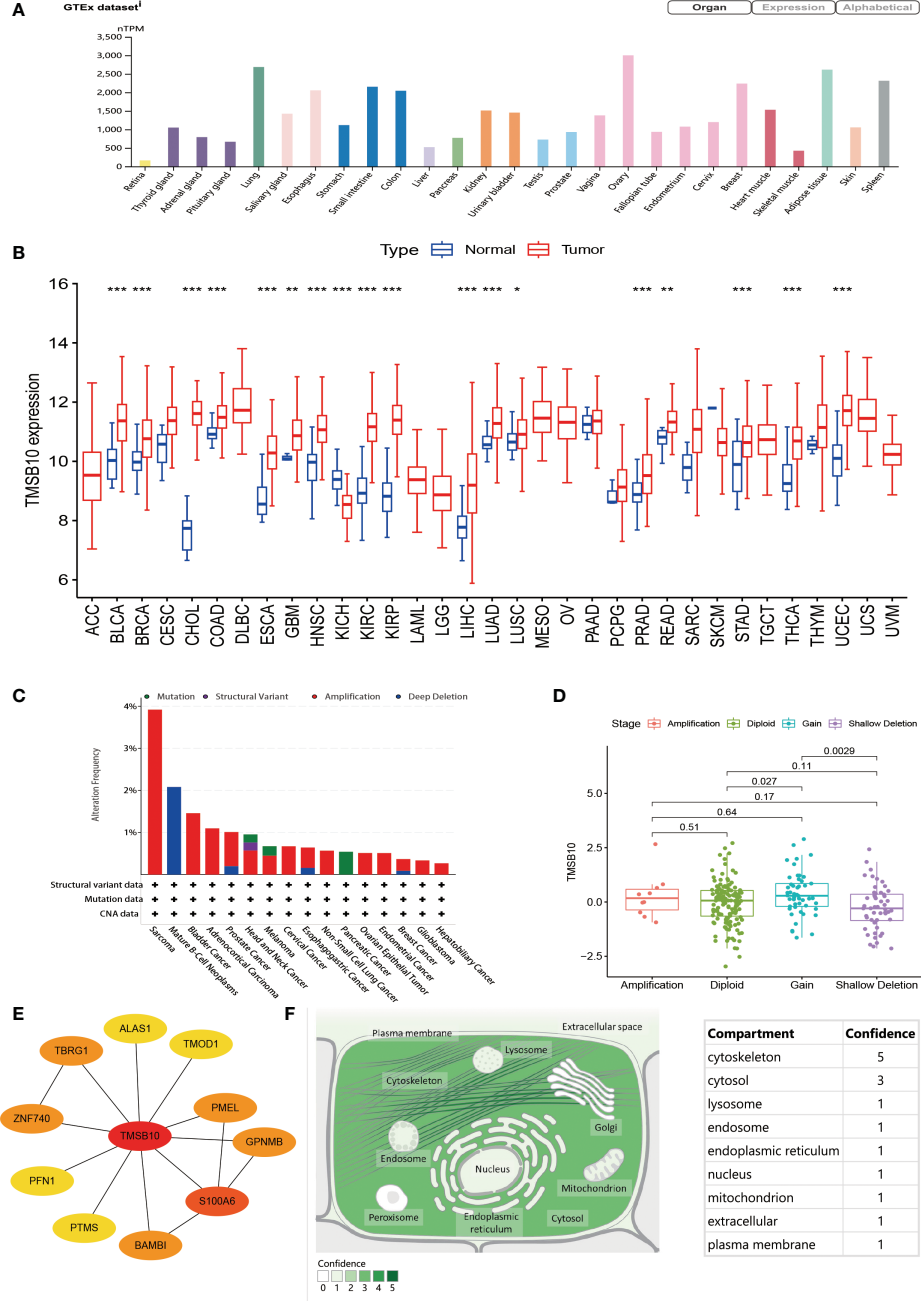
General information of TMSB10. **(A)** Expression level of TMSB in different organ. **(B)** Comparisons of TMSB10 expression levels between tumor and normal tissues. **(C)** Landscapes of TMSB10 in pan-cancer. **(D)** TMSB10 expression of different gene mutations in Sarcoma. **(E)** Protein-protein interaction for TMSB10. **(F)** Subcellular locations of TMSB10. *P<0.05; **P<0.01; ***P<0.001.

By integrating GTEx database, the top three normal tissues with high expression of TMSB10 are ovary, lung, and adipose tissue (**Figure 1A**). By integrating TCGA database, the expression level of TMSB10 in tumor tissues was higher than the corresponding normal tissues, and the result showed that TMSB10 is highly expressed in 15 tumor types: BLCA, BRCA, CHOL, COAD, ESCA, GBM, HNSC, KIRC, KIRP, LUAD, LUSC, READ, STAD, THCA and UCEC (all P<0.05). By contrast, TMSB10 is lowly expressed in 3 tumor types: KICH、LIHC and PRAD (all P<0.05). (**Figure 1B**)

### Gene alteration levels of TMSB10 in pan-cancer

In view of the abnormal expression of TMSB10 in pan-cancer, we speculate that this phenomenon may be related to the genetic alteration of TMSB10. The result showed that Patients with Sarcoma had the highest “Amplification” frequency of TMSB10 genetic alteration (>3%). Therefore, we further analyzed the expression of TMSB10 in the Sarcoma “Amplification” group, “Diploid” group, “Gain” group and “Shallow Deletion” group, and found that the expression of TMSB10 in the “Diploid” group was significantly higher than “Shallow Deletion” group (**Figure 1D**). The “Structural Variant” were found in the Mature B-cell Neoplasms with a frequency of about 2%. The “Mutation” type of copy number alteration (CNA) was only found in Pancreatic cancer (**Figure 1C**).

### Interaction and subcellular locations of TMSB10

By searching the ComPPI database, TMSB10 participates in several protein-protein interactions. The most closely related to S100A6 (**Figure 1E**). By querying the Human Protein Atlas (HPA), the subcellular localization of TMSB10 was mainly located in the cytoskeleton, then followed by cytosol (**Figure 1F**).

### Prognosis roles of TMSB10 in pan-cancer

**Figure 2:**
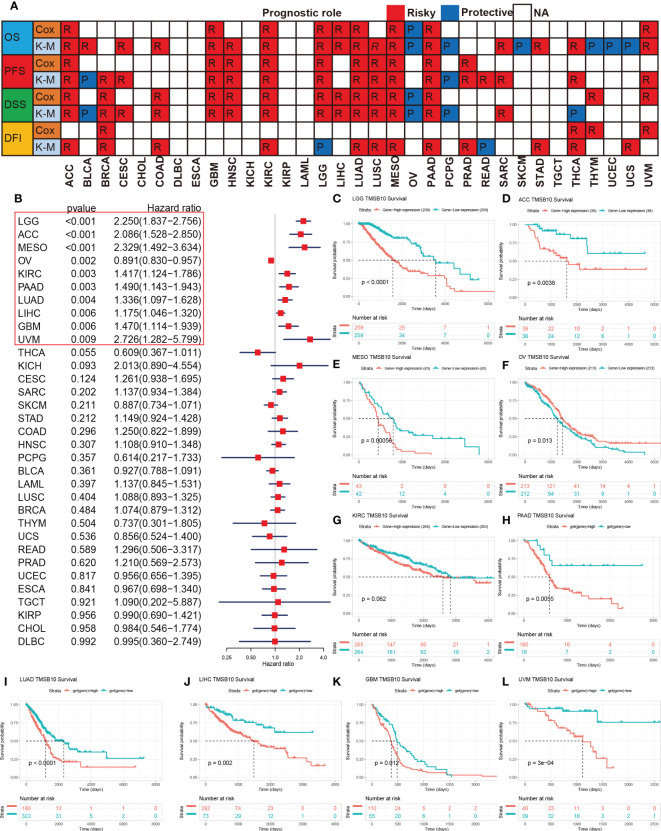
Prognosis roles of TMSB10 in pan-cancer. **(A)** Correlations of TMSB10 with OS, PFS, DSS and DFI using cox regression and Kaplan-Meier methods in pan-cancer. **(B)** Forest indicated the correlations of TMSB10 with OS in pan-cancer. **(C-L)** Kaplan-Meier survival curve of OS in LGG, ACC, MESO, OV, KIRC, PAAD, LUAD, LIHC, GBM AND UVM.

To further investigate the predictive potential of TMSB10 in pan-cancer, we compared and analyzed four prognostic indicators of 33 cancers, which contained Overall Survival (OS), Progression-Free Survival (PFS), Disease-Specific Survival (DSS) and Disease-Free Interval (DFI). Heat map results showed that TMSB10 was highly correlated with the prognosis of multiple cancers. It is a risk factor for poor prognosis for ACC, BRCA, CESC, COAD, GBM, HNSC, KIRC, LGG, LIHC, LUAD, LUSC, MESO, PAAD, PRAD, SARC, STAD, THCA, THYM, and UVM. In contrast, TMSB10 is a protective factor for BLCA, OV, PCPG, SKCM, and UCEC, and it is not associated with CHOL, DLBC, ESCA and TGCT (**Figure 2A**). The Forest map results showed that downregulation of TMSB10 expression could prolong the OS of some tumors: LGG (HR=2.250 [95%CI, 1.837-2.756], P<0.001), ACC (HR=2.086 [95%CI, 1.528-2.850], P<0.001), MESO (HR=2.329 [95%CI, 1.492-3.634], P<0.001), KIRC (HR=1.417 [95%CI, 1.124-1.786], P=0.003), PAAD (HR=1.490 [95%CI, 1.143-1.943], P=0.003), LUAD (HR=1.336 [95%CI, P=0.004), LIHC (HR=1.175 [95%CI, 1.046-1.320], P=0.006), GBM (HR=1.470 [95%CI, 1.114-1.939], P=0.006), UNM (HR=2.726 [95%CI, 1.282-5.799], P=0.004). Upregulation of TMSB10 expression could shorten the OS in OV (HR=0.891 [95%CI, 0.830-0.957], P=0.002) (**Figure 2B**, **Figure S1**). In addition, we analyzed Kaplan-Meier curves of these tumors which have statistical significance, found that low expression of TMSB10 is an important favorable prognosis marker in many tumors, such as LGG, ACC, MESO, PAAD, LUAD, LIHC, GBM (all P<0.05). In Comparison, we also found that high TMSB10 expression in OV had a favorable prognosis. Therefore, we suppose that TMSB10 has an important value in predicting the prognosis of many cancers (**Figures 2C–L**).

### Gene set variation analysis identified the correlations of TMSB10 with immune response

**Figure 3:**
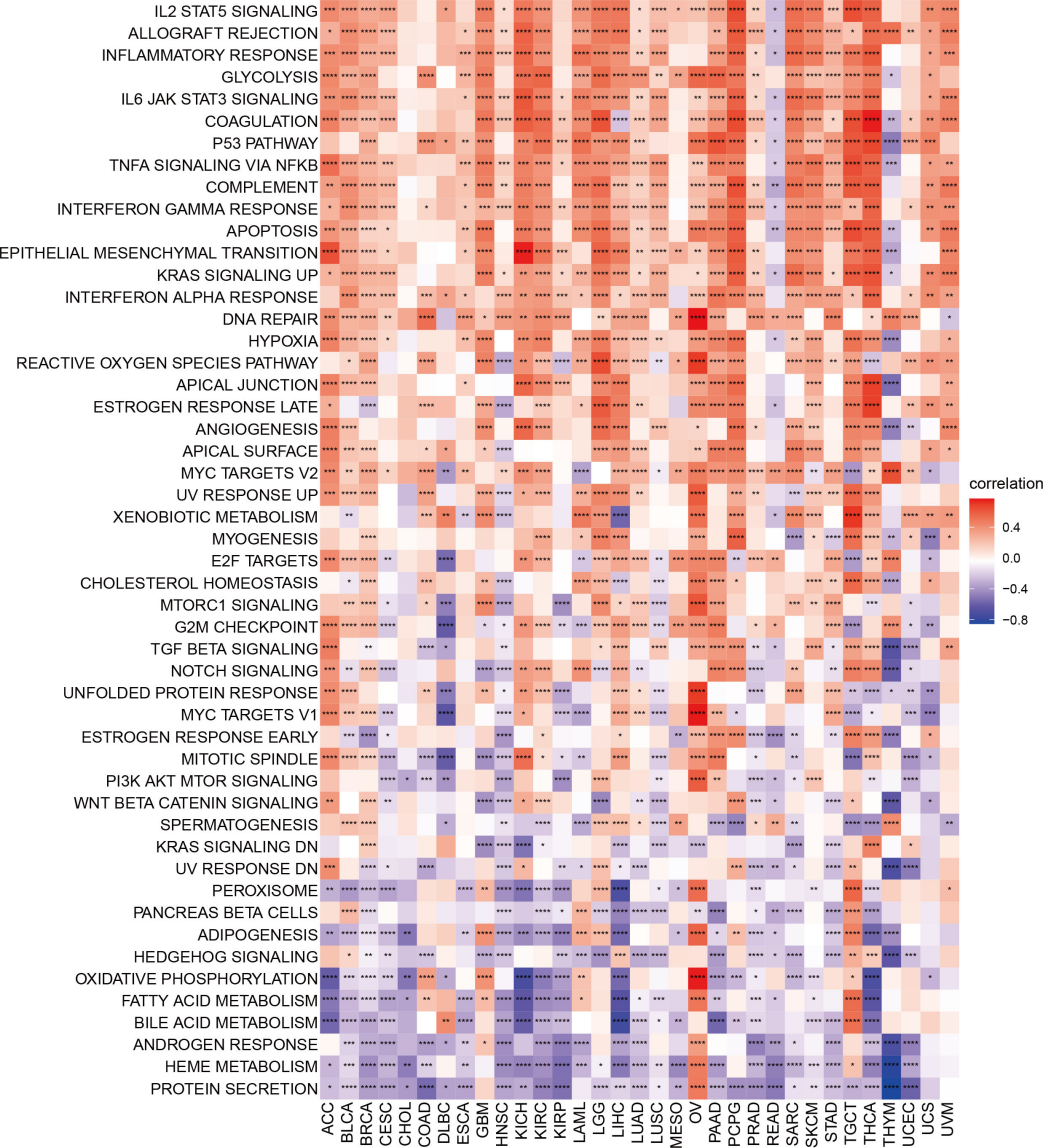
Gene set variation analysis of TMSB10 in pan-cancer *P<0.05; **P<0.01; ***P<0.001; ****P<0.0001.

To further explore the biological processes of TMSB10 in cancer initiation and development, we performed Gene Set Variation Analysis (GSVA) on 33 cancers to evaluate the relationship between TMSB10 and 50 common cancer signaling pathways, tumor immune and inflammatory responses. The results showed the immune and inflammation-related pathways:IL2-STAT5-signaling, Allograft-rejection, Inflammatory Response, IL6-JAK-STAT3-signaling, TNFA-signaling-via-NFKB, IFN-α-Response and IFN-γ-Response, and TMSB10 was significantly and positively enriched in various tumors, especially in BLCA, GBM, KICH, KIRC, PCPG and THCA. These results suggest that TMSB10 may be closely related to the tumor immune microenvironment and immune Response. Our results also showed that TMSB10 was positively enriched in most OV and TGCT signaling-pathways, immune and inflammatory reactions, while negatively enriched in READ and THYM. In addition, we found that TMSB10 was positively correlated with Epithelial Mesenchymal Transformation (EMT) in a variety of tumors: ACC, BLCA, BRCA, GBM, KICH, KIRC, LGG, LIHC, LUSC, PAAD, PCPG, SARC, SKCM, TGCT, THCA and SKCM, suggesting that TMSB10 may play an important role in tumor invasion and migration (**Figure 3**). TMSB10 may exert its function in the development and progression of cancer by regulating tumor microenvironment and mediating tumor-related immune and inflammatory responses.

### Tumor microenvironment and immune infiltration Levels of TMSB10 in pan-cancer

**Figure 4:**
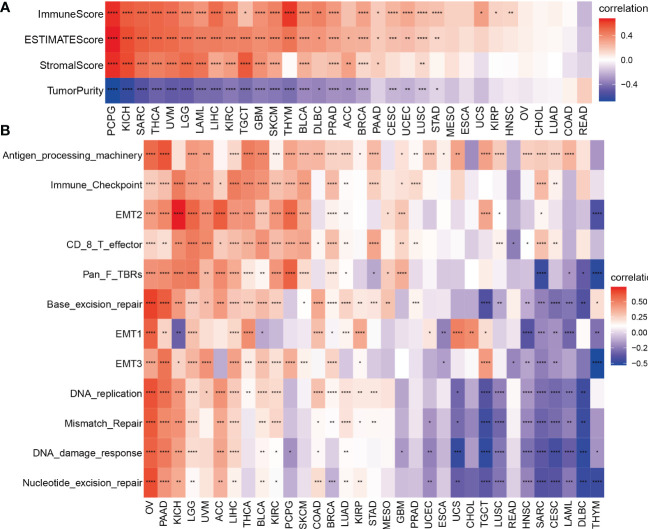
Tumor microenvironment analysis of TMSB10 in pan-cancer. **(A)** ESTIMATE method. **(B)** enrichment score method *P<0.05; **P<0.01; ***P<0.001; ****P<0.0001.

**Figure 5:**
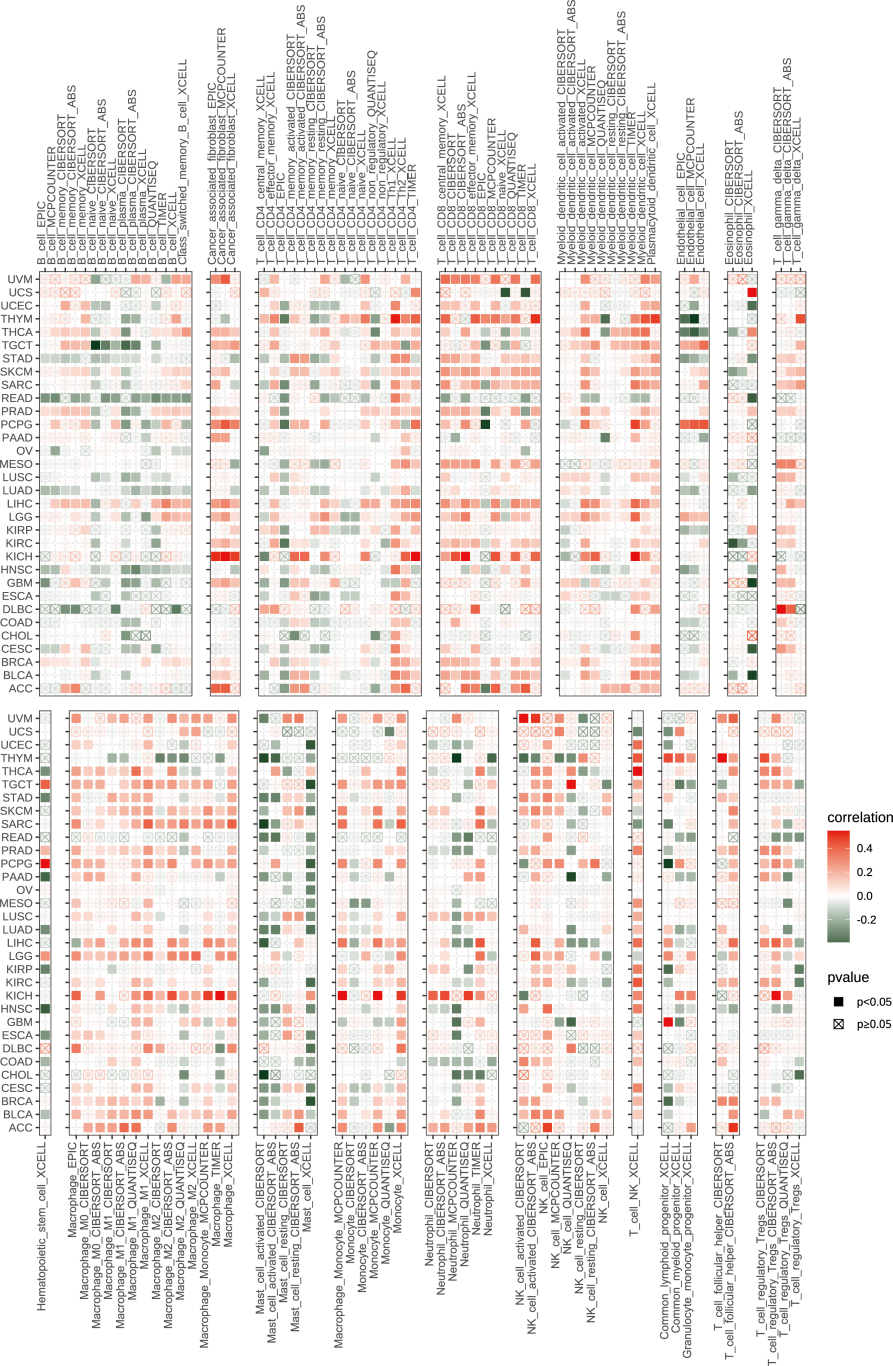
Immune infiltration levels of TMSB10 in different immune cells.

Our previous results showed that TMSB10 was associated with tumor immune microenvironment. Therefore, we analyzed the relationship between TMSB10 and tumor immune microenvironment by ESTIMATE score and Enrichment score, and the results verified that TMSB10 was positively correlated with the level of immune cell infiltration in a variety of tumors, especially in KICH、UVM、LGG and LIHC (**Figures 4A, B**). The relationship between TMSB10 expression and various immune cell infiltration in pan-cancer was explored by TIMER2.0 database. The results showed that TMSB10 was positively correlated with some immune cells, such as T cells gamma and delta, CD4_Th1 and Th2, macrophage M1 and M2, CD8^+^ T cells, endothelial cells (Endo), and eosinophils (Eos) (**Figure 5**). We also explored the correlations of TMSB10 with cell-specific markers in pan-cancer. The **Figure S2** presented the results, and we found that TMSB10 was positively associated with these cell-specific markers in LIHC, THCA, TGCT, KICH, LGG, PCPG, SARC, GEM, BLCA, SKCM, and BRCA. Some markers CD14 (monocyte), CD3D (CD4+T cells), CD3E (CD8+T cells), CD68 (macrophage), CST3 (myeloid cells), GNLY (NK cells), KRT18 (epithelial cells), and NKG7 (NK cells) were positively associated with TMSB10 in all cancers.

### Correlations of TMSB10 with immune regulators, TMB and MSI

**Figure 6:**
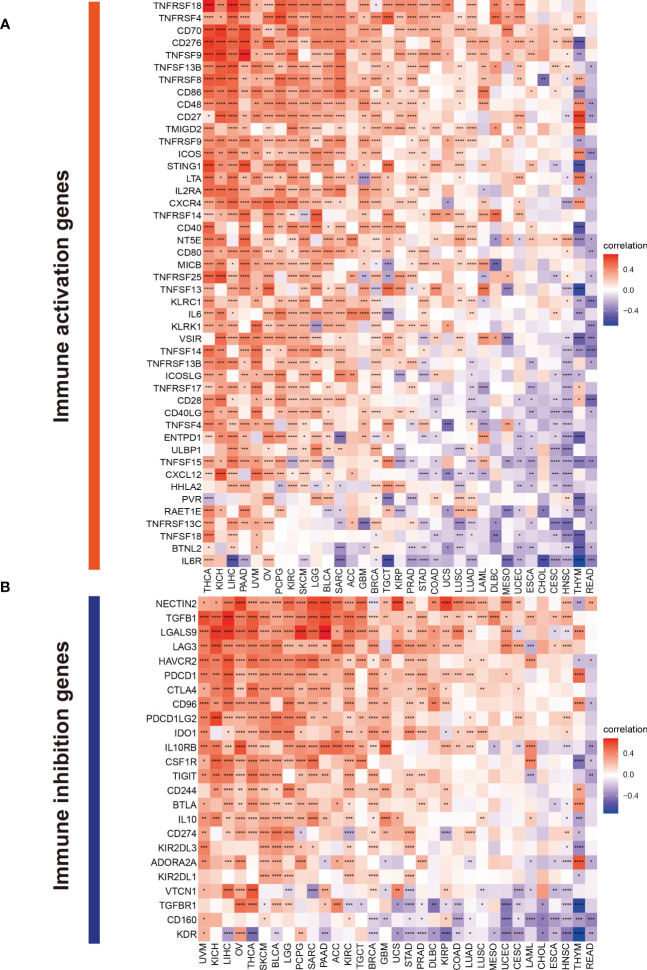
Correlations of TMSB10 with immune genes. **(A)** immune activation genes. **(B)** immune inhibition genes *P<0.05; **P<0.01; ***P<0.001; ****P<0.0001.

**Figure 7:**
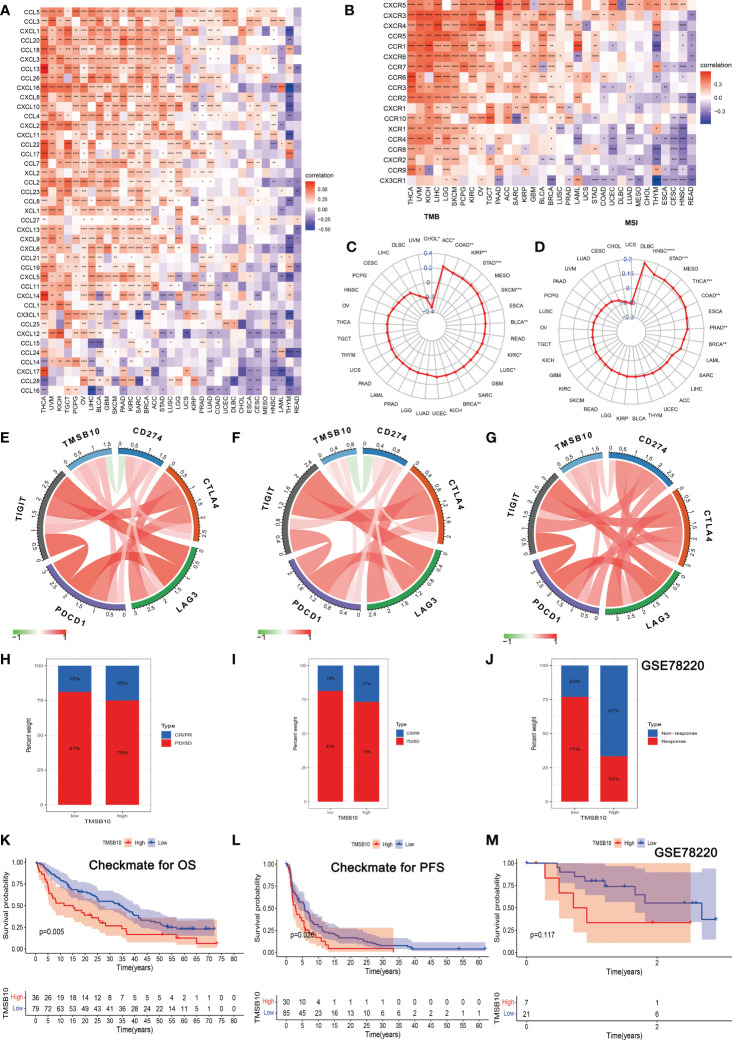
Correlations of TMSB10 with chemokines and immune checkpoint key genes and its effect on immunotherapy. **(A, B)** Associations between TMSB10 and chemokines and its receptors (*P<0.05, **P<0.01, ***P<0.001). **(C, D)** Correlations of TMSB10 with TMB and MSI levels. **(E-G)** Associations between TMSB10 with immune checkpoint key genes in KIRC, KICH, and Melanoma. **(H-K)** Effects of TMSB10 expression on anti-PD-L1 treatment responses and OS in Checkmate cohorts. **(I-L)** Effects of TMSB10 expression on anti-PD-L1 treatment response and PFS in Checkmate cohorts. **(J, M)** Effects of TMSB10 expression on anti-PD-L1 treatment response and OS in GSE78220 cohorts. *P<0.05; **P<0.01; ***P<0.001; ****P<0.0001.

The Spearman correlation analysis was performed to know the relationship between TMSB10 and 46 immunoregulatory genes (**Figure 6A**). The results showed that TMSB10 was positively correlated with various tumor immune activation genes, especially in THCA, KICH, PAAD, OV, PCPG, KIRC and LGG, but negatively correlated with THYM and READ. We also found that TMSB10 was positively correlated with multiple tumor immune inhibition genes, especially in UVM, KICH, LIHC, OV, THCA, SKCM, BLCA, LGG, and PCPG, while READ was negatively correlated (**Figure 6B**). In conclusion, TMSB10 as a “dual-role” is related to both immune activation gene and immune inhibition genes in KICH, THCA and LGG. The interaction of chemokines and their receptors controls the targeted migration of various immune cells, clears the source of infection, promotes wound healing, and destroys the function of abnormal proliferating cells. Our results showed that TMSB10 expression was positively correlated with the expression of multiple chemokines and their receptors, such as THCA, UVM and KICH (**Figures 7A, B**). In order to further understand the role of TMSB10 in predicting the efficacy of Immune Checkpoint Inhibitor (ICI), we used Tumor Mutation Burden (TMB) and Microsatellite Instability (MIS) to predict the relationship between TMSB10 and the efficacy of immunotherapy. The results showed that the expression of TMSB10 in COAD, KIRP, STAD, SKCM, BLCA and BRCA was positively correlated with TMB, and negatively correlated in DLBC, LIHC and CESC (**Figure 7C**). In addition, the expression of TMSB10 was positively correlated with MSI in HNSC, STAD, THCA, COAD, PRAD and BRCA, and negatively correlated in CHOL, CESC, LUAD, and UVM (**Figure 7D**). In addition, we found that TMSB10 could effectively predicted the effect of anti-PDL1 (PDCD1), anti-CTLA4 and anti-TIGIT immunotherapy in KIRC, KICH and MESO, and the expression of TMSB10 is positively correlated with immune cell infiltration (**Figures 7E–G**). These results indicated that TMSB10 may be associated with immune regulation in some cancer.

### TMSB10 predicts immunotherapy response

In the Checkmate cohort, the efficacy of immune checkpoint inhibitors (anti-PD-L1) in the TMSB10 high expression group was worse than that in the low expression group (**Figures 7H, I**), and the OS and PFS in the TMSB10 high expression group were also lower (**Figures 7K, L**). However, in the GSE78220 cohort, although the efficacy of anti-PD-L1 therapy was worse in the TMSB10 high expression group, there was no significant effect on OS (**Figures 7J, M**). TMSB10 may be used as an effective marker to predict the efficacy of immune checkpoint inhibitors in some cancer.

### Effect of TMSB10 on chemotherapy sensitivity

The relationship between gene expression and the efficacy of chemotherapy was reflected in the immune infiltration correlation plot. We selected the top 6 small molecule compounds with chemotherapy resistance/sensitivity as examples, and the results showed that the expression of TMSB10 was positively correlated with some chemotherapy resistance, especially in Sorafenib, LGK 974, Vorinostat, AZD5991, TAF1 and AZD1208. However, TMSB10 was positively associated with sensitivity to some chemotherapy, especially in ZM447439, BI-2536, JQ1, Nu7441, Tozasertib and Dasatinib (**Figure S3**, **Table S2**).

### TMSB10 promotes glioma progression by regulating PD-L1 expression *via* IL6/JAK/STAT3 signaling pathway

**Figure 8:**
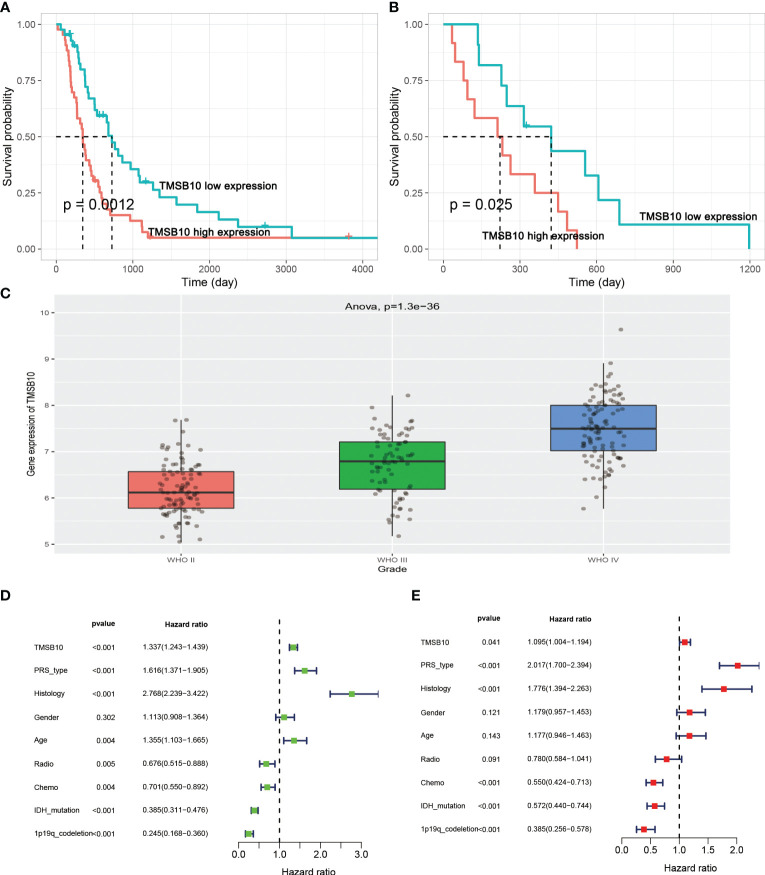
TMSB10 is an independent prognosis factor in glioma. **(A, B)** High-expressed TMSB10 is associated with poor OS and PFS**(C)** TMSB10 increased with grade. **(D, E)** Univariate and multivariate cox regression indicated that TMSB10 was associated with prognosis in glioma.

We further explored the function role of TMSB10 in glioma through cell experiments. In CGGA, high-expressed TMSB10 was associated with poor OS (**Figure 8A**) and PFS (**Figure 8B**). Next, we compared the TMSB10 expression level among different grade, and we found glioma patients with advanced grade had higher TMSB10 expression (**Figure 8C**, *P*=1.3e-36). Finally, univariate, and multivariate cox regression showed that TMSB10 was an independent prognosis factor for glioma (univariate: HR=1.337, 95%CI: 1.243-1.439, P<0.001, **Figure 8D**; Multivariate: HR=1.095, 95%CI: 1.04-1.194, P=0.041, **Figure 8E**).

**Figure 9:**
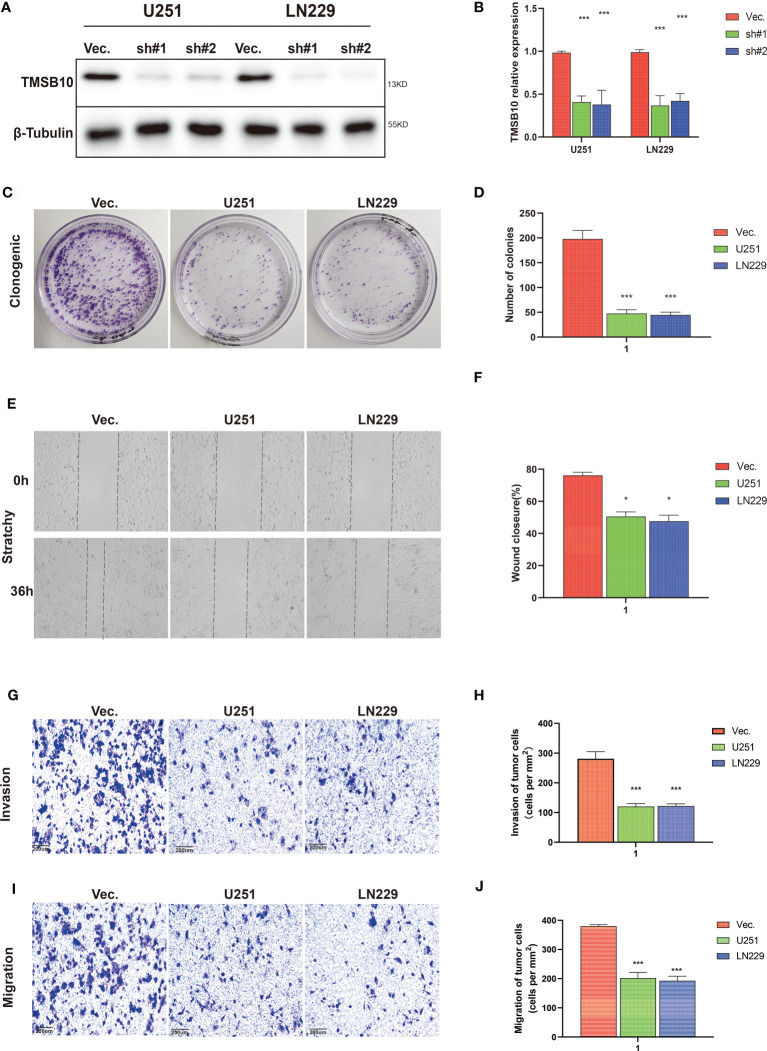
TMSB10 involves in glioma progression. **(A, B)** Establishment of TMSB10 knock out cells. **(C, D)** Low expression of TMSB10 inhibited the clonogenic formation ability of glioma cells. **(E , F)** Low expression of TMSB10 inhibited migration of glioma cells. **(G–J)** Low expression of TMSB10 inhibits the invasion and migration of glioma cells. *P<0.05; ***P<0.001.

**Figure 10:**
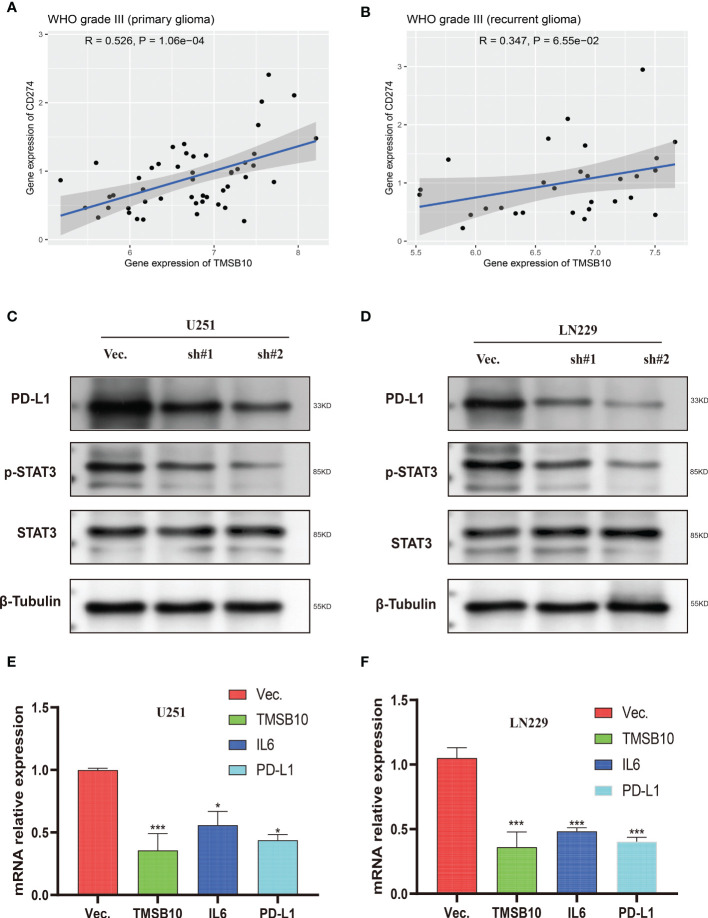
Low expression of TMSB10 inhibited PD-L1 expression *via* regulating STAT3 signaling. **(A, B)** TMSB10 was positively associated with PD-L1 in primary and recurrent glioma. **(C, D)** Western blot indicated PD-L1 and p-STAT3 were down-regulated after TMSB10 inhibition in glioma cells. **(E, F)** q-PCR indicated IL6 and PD-L1 were low expressed after TMSB10 inhibited. *P<0.05; ***P<0.001.

Then, we built glioma cells of TMSB10 low expression (**Figures 9A, B**). We found low expression of TMSB10 inhibited clonogenic formation ability, invasion, migration *in vitro* (**Figures 9C–J**). Furthermore, the correlation analyses indicated that TMSB10 were positively associated with PD-L1 expression level in primary and recurrent glioma (**Figures 10A, B**). The pathways enrichment analysis indicated TMSB10 was positively associated with IL6/JAK/STAT3 signaling pathway (**Figure S4**), and we also found TMSB10 was positively associated with IL-6 in patients with primary/recurrent glioma (**Figures S5A-H**, grade III: r=0.46, *P*<0.001, grade IV: r=0.317, *P*=0.003). Western blot indicated that PD-L1 and p-STAT3 expression levels were significantly down-regulated after TMSB10 knock down (**Figures 10C, D**). qPCR also indicated that mRNA levels of PD-L1 and IL6 were down-regulated after TMSB10 knock down (**Figures 10E, F**). Furthermore, we found PD-L1 expression and p-STAT3 levels were increased in TMSB10-knock down cells using IL-6 stimulating for 36 hours (**Figures S6A–C**). TMSB10 may involve glioma immune regulation progression by promoting PD-L1 expression levels *via* activating STAT3 signaling pathway.

## Discussion

Immune checkpoint inhibitors (ICIs) play an important role in the maintenance of tolerance and tissue damage caused by immune response by regulating the number and function of antigen-specific T cells. The immune checkpoints mainly include programmed death-1 (PD-1), cytotoxic T lymphocyte associated antigen 4 (CTLA-4), T cell Immunoglobulin domain and Mucindomain-3 (TIM-3), which can induce immunosuppressive responses of T cells and promote T cell failure by binding with corresponding ligands on the surface of tumor cells. Prompting tumor cells to evade the immune system’s surveillance (13, 14). At present, the monoclonal antibodies that block the interaction of PD-L1/PD-1 have been clinically approved for many solid cancer immunotherapy, especially refractory or advanced tumors. However, although antibody drugs have been used wildly in the clinical cure, the curative effective is still not satisfied enough, the effective rate of PD-1/PD-L1 inhibitor treatment is only 20%-40% in many solid tumors (15, 16), which emphasizes the necessity of developing new immune checkpoints to predict the prognosis of cancer immunotherapy. In our study, we proved that TMSB10 could become a hopeful prognosis biomarker in pan-cancer, especially in the response of cancer immunotherapy and chemotherapy in the future.

According to our study, the expression level of TMSB10 is not only different in normal tissues, but it was higher in many tumor tissues by integrating GTEx and TCGA database. Our result showed that TMSB10 is highly expressed in 15 tumor types and lowly expressed in 3 tumor types. Our findings are consistent with most previous studies that have shown that overexpression of TMSB10 is closely related to the occurrence and development of gastric cancer, breast cancer, bladder cancer and hepatocellular carcinoma (2, 17–19). In the Landscapes, it was found that sarcoma had the highest gene alteration frequency of TMSB10 in pan-cancer and we analyzed the expression of TMSB10 in different gene mutations in sarcomas, the result showed that Diploid and Shallow Deletion were two of the highest incidence of mutation types.

Then, we analyzed the clinical prognostic with the expression level of TMSB10 in 33 cancers. Our results showed that TMSB10 was highly correlated with the prognosis of multiple cancers. High expression of TMSB10 could predict a poor prognosis in 19 cancers, and low expression of TMSB10 could predict a positive prognosis in 5 cancers. Our results also showed that downregulation of TMSB10 expression could prolong the OS of 9 tumors, and upregulation of TMSB10 expression could shorten the OS in 1 tumor. Upregulation of TMSB10 is involved in a variety of signaling pathways related to tumor invasion and metastasis, leading to unsatisfied survival rate of patients. Therefore, we suppose that TMSB10 has an important value in predicting the prognosis of many cancers.

In recent years, tumor microenvironment has received more and more attention because it plays a key role in tumor immune escape, distant metastasis, treatment resistance and targeted therapy response (20, 21). We explored the relationship between tumor microenvironment and TMSB10 expression in pan-cancer. The GSVA results showed that TMSB10 expression closely related to immune and inflammation-related pathways in many tumors and positively correlated with EMT in a variety of tumors, and TMSB10 was positively correlated with the level of immune cell infiltration in a variety of tumors, especially in KICH、UVM、LGG and LIHC. TMSB10 was positively correlated with the infiltration levels of various immune cells, such as T cells, B cells, CD8+T cells, endothelial cells (Endo), and eosinophils (Eos). These results suggested that TMSB10 is associated with tumor immune microenvironment.

Next, we hope to know the relationship between TMSB10 expression and immunoregulatory genes. Our results showed that upregulate TMSB10 expression was related with immune activation genes in 7 tumors, and upregulate TMSB10 expression was related with immune inhibition genes in 9 tumors. In addition, TMSB10 as a “dual-role” is related to both immune activation gene and immune inhibition genes in KICH, THCA and LGG. Our results showed that TMSB10 expression was positively correlated with the expression of multiple chemokines and their receptors in THCA, UVM and KICH. We used the TMB and MIS to predict the relationship between TMSB10 and the efficacy of immunotherapy. The results showed that the expression of TMSB10 in COAD, KIRP, STAD, SKCM, BLCA and BRCA was positively correlated with TMB, and negatively correlated in DLBC, LIHC and CESC. And the expression of TMSB10 was positively correlated with MSI in HNSC, STAD, THCA, COAD, PRAD and BRCA, and negatively correlated in CHOL, CESC, LUAD, and UVM. In addition, we found that TMSB10 could effectively predicted the effect of anti-PDL1, anti-CTLA4 and anti-TIGIT immunotherapy in KIRC KICH, and MESO, and the expression of TMSB10 is positively correlated with immune cell infiltration. To validate our findings, we performed the experiments *in vitro* and found low expression of TMSB10 inhibited clonogenic formation ability, invasion, and migration in glioma cells. Furthermore, TMSB10 may involve glioma immune regulation progression by promoting PD-L1 expression levels *via* activating STAT3 signaling pathway in glioma cells. Therefore, we hypothesized that TMSB10 could be an effective biomarker to predict the efficacy of immune checkpoint inhibitors in pan-cancer.

Finally, we verified the expression of TMSB10 could reflect immunotherapy and chemotherapy responses in some tumors, especially in KIRC, KICH and MESO. Our results showed that the efficacy of anti-PD-L1 in the TMSB10 high expression group was worse than that in the low expression group in KIRC and KICH, and the OS and PFS in the TMSB10 high expression group were also lower. However, in the GSE78220 cohort, although the efficacy of anti-PD-L1 therapy was worse in the TMSB10 high expression group, there was no significant effect on OS. We further found that the expression of TMSB10 has a closely relationship with chemotherapy resistance and sensitivity.

Previous study also explored the function roles of TMSB10 in pan-cancer (22). Both previous and our study had bioinformatics analyses and experiment validations. However, there are remarkedly differences between previous study and our study. For bioinformatics analyses, previous study only presented the pan-cancer expression pattern and biological and immunomodulatory function of TMSB10, and most of results focus on glioma. But our study included prognosis analyses, pathway enrichment, tumor microenvironment and immune infiltration, immune checkpoints, immune activation genes, immune inhibition genes, chemokines and their receptor genes, tumor mutation burden, and microsatellite instability in pan-cancer. We also explored the effect of TMSB10 on immunotherapy in real world cohort data. We showed a huge landscape in pan-cancer, which is completely different from previous study. For experiments validation, we admitted that previous study presented more details, but we have different findings for TMSB10 in glioma. Previous study explored the TMSB10 promoted glioma progression *via* YAP1/AKT/ERK1/2, but we explored the TMSB10 promoted glioma progression *via* IL6/JAK/STAT3 signaling pathway. But we both proved that TMSB10 can be a potential immunotherapy target point in glioma. Gene regulation is a complex process, and we provide a different view and enriched the molecular mechanism in glioma.

There are several limitations to our study. First, the association with protein levels needs to be tested *in vivo*. Second, validation can be performed with other public datasets to further support our current findings. Third, anti-tumor activity can be measured by targeting TMSB10, and the role of TMSB10 in immune checkpoints and its effect on chemotherapy sensitivity can be validated in conjunction with more clinical trials.

In conclusion, we performed a comprehensive evaluation of TMSB10, revealing its potential role as a prognostic indicator for patients in immunomodulatory and therapeutic efficacy. TMSB10 may become a novel target for tumor immunotherapy and chemotherapy.

## Data availability statement

The original contributions presented in the study are included in the article/**Supplementary Material**. Further inquiries can be directed to the corresponding author.

## Ethics statement

Ethical approval was not provided for this study on human participants because Data of Human was from publica data. The ethics committee waived the requirement of written informed consent for participation.

## Author contributions

ZL designed this study and directed the research group in all aspects, including planning, execution, and analysis of the study. YZ drafted the manuscript. YL and NL collected the data. ZL provided the statistical software, performed the data analysis. YT and NL arranged the Figures and Tables. ZL and LS revised the manuscript. All authors contributed to the article and approved the submitted version.

## Supplementary material

Supplementary Figure 1 The correlations of TMSB10 with PFI **(A)**, DFI **(B)** and DSS **(C)** in cancers.

Supplementary Figure 2 Correlations of TMSB10 with cell-specific markers in pan-cancer.

Supplementary Figure 3 Correlations of TMSB10 with Chemotherapy sensitivity. **(A)** Top 6 compounds showing resistant to TMSB10. **(B)** Top 6 compounds showing sensitivity to TMSB10.

Supplementary Figure 4 Pathways enrichment analysis of TMSB10.

Supplementary Figure 5 TMSB10 was positively associated with IL-6 in primary and recurrent glioma **(A-H)**.

Supplementary Figure 6 IL-6 affect the PD-L1 **(A)** and p-STAT3 levels **(B, C)**.
