# Correction: Pan-cancer analysis identifies the correlations of Thymosin Beta 10 with predicting prognosis and immunotherapy response

**DOI:** 10.3389/fimmu.2025.1676745

**Published:** 2025-08-12

**Authors:** Zhanzhan Li, Yanyan Li, Yifu Tian, Na Li, Liangfang Shen, Yajie Zhao

**Affiliations:** ^1^ Department of Oncology, Xiangya Hospital, Central South University, Changsha, Hunan, China; ^2^ Department of Nursing, Xiangya Hospital, Central South University, Changsha, Hunan, China; ^3^ Department of Pathology, Xiangya Hospital, Central South University, Changsha, Hunan, China; ^4^ Department of Nuclear Medicine, Xiangya Hospital, Central South University, Changsha, Hunan, China; ^5^ National Clinical Research Center for Geriatric Disorders, Xiangya Hospital, Central South University, Changsha, Hunan, China

**Keywords:** Keywords: TMSB10, pan-cancer, immunotherapy, prognosis, immune infiltration

In the published article, there were errors in the **Funding** statement. The Funding statement is incomplete. The correct **Funding** statement appears below.

“This study was supported by the National Natural Science Foundation of China (LZZ: No.82003239), Hunan Provincial Natural Science Foundation of China (LZZ: No.2023JJ30907), and National key clinical specialty (SLF: 2209090720).”

The original version of this article has been updated.

