# Supplementary figures and images for: Pan-cancer analysis identifies the correlations of Thymosin Beta 10 with predicting prognosis and immunotherapy response
Source: Front Immunol. 2023 May 18;14:1170539. doi: 10.3389/fimmu.2023.1170539 (PMC10232749; doi:10.3389/fimmu.2023.1170539)

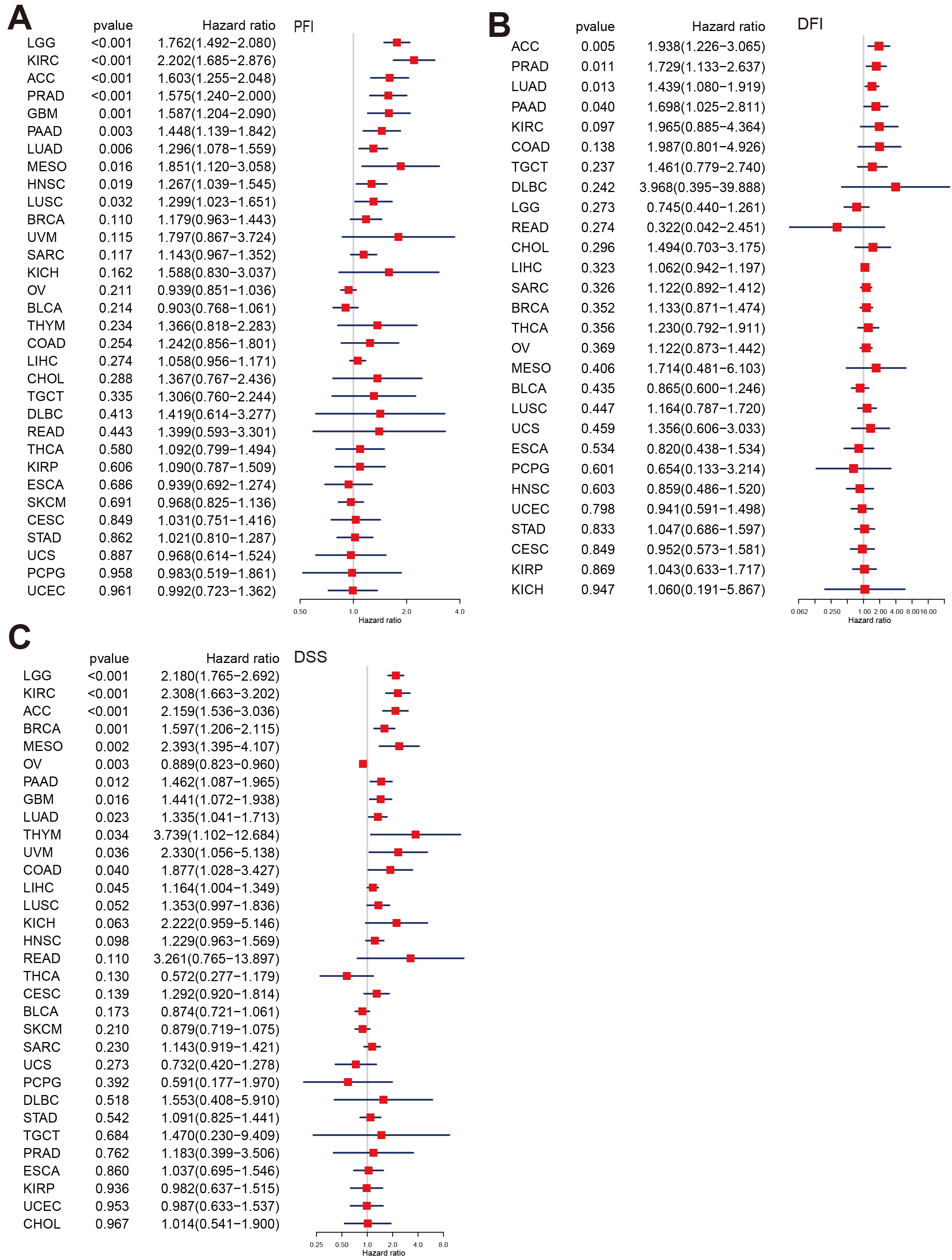

Supplement: Supplementary Figure 1 — The correlations of TMSB10 with PFI (A), DFI (B) and DSS (C) in cancers. [file Image1.tif]

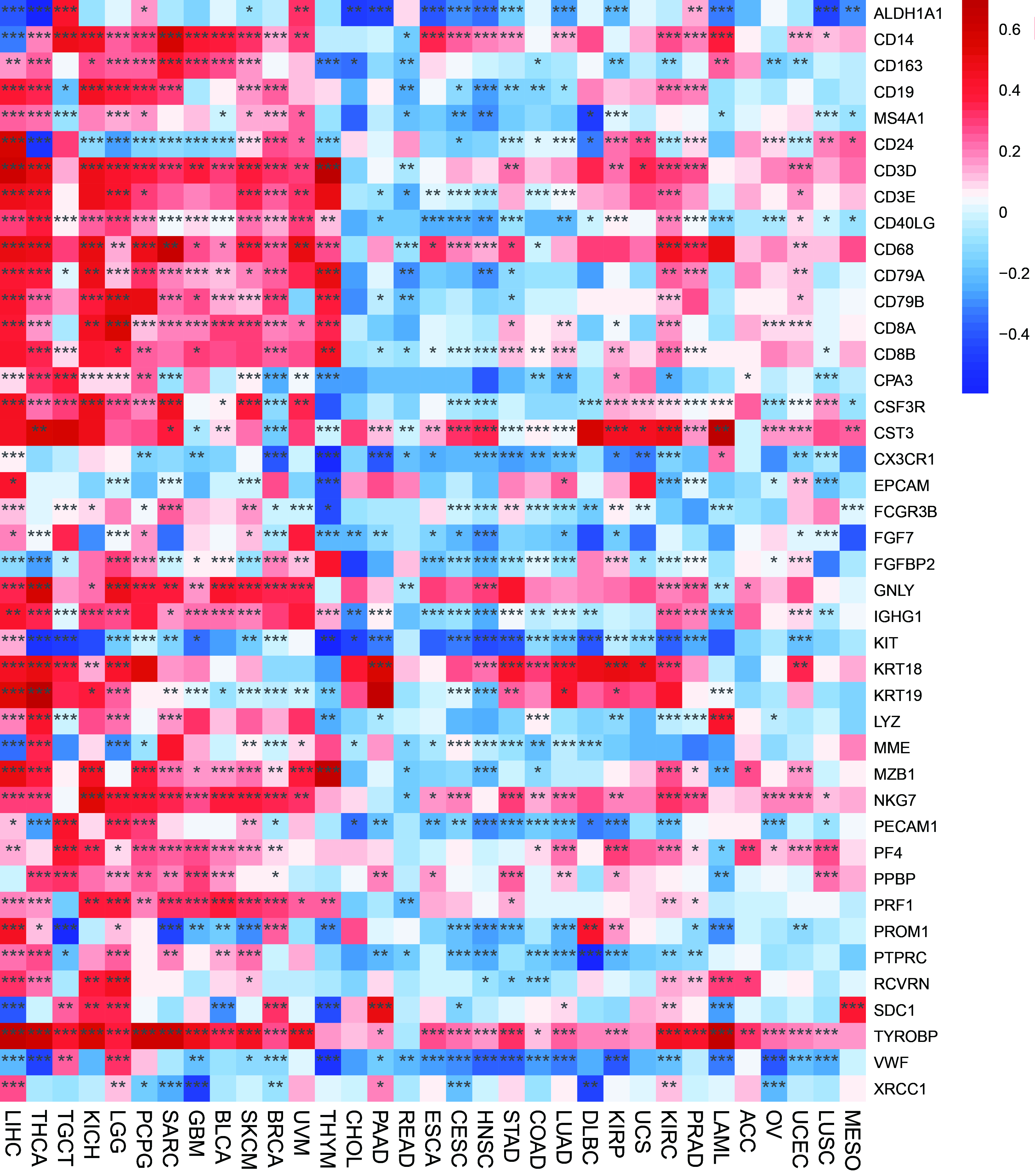

Supplement: Supplementary Figure 2 — Correlations of TMSB10 with cell-specific markers in pan-cancer. [file Image2.tif]

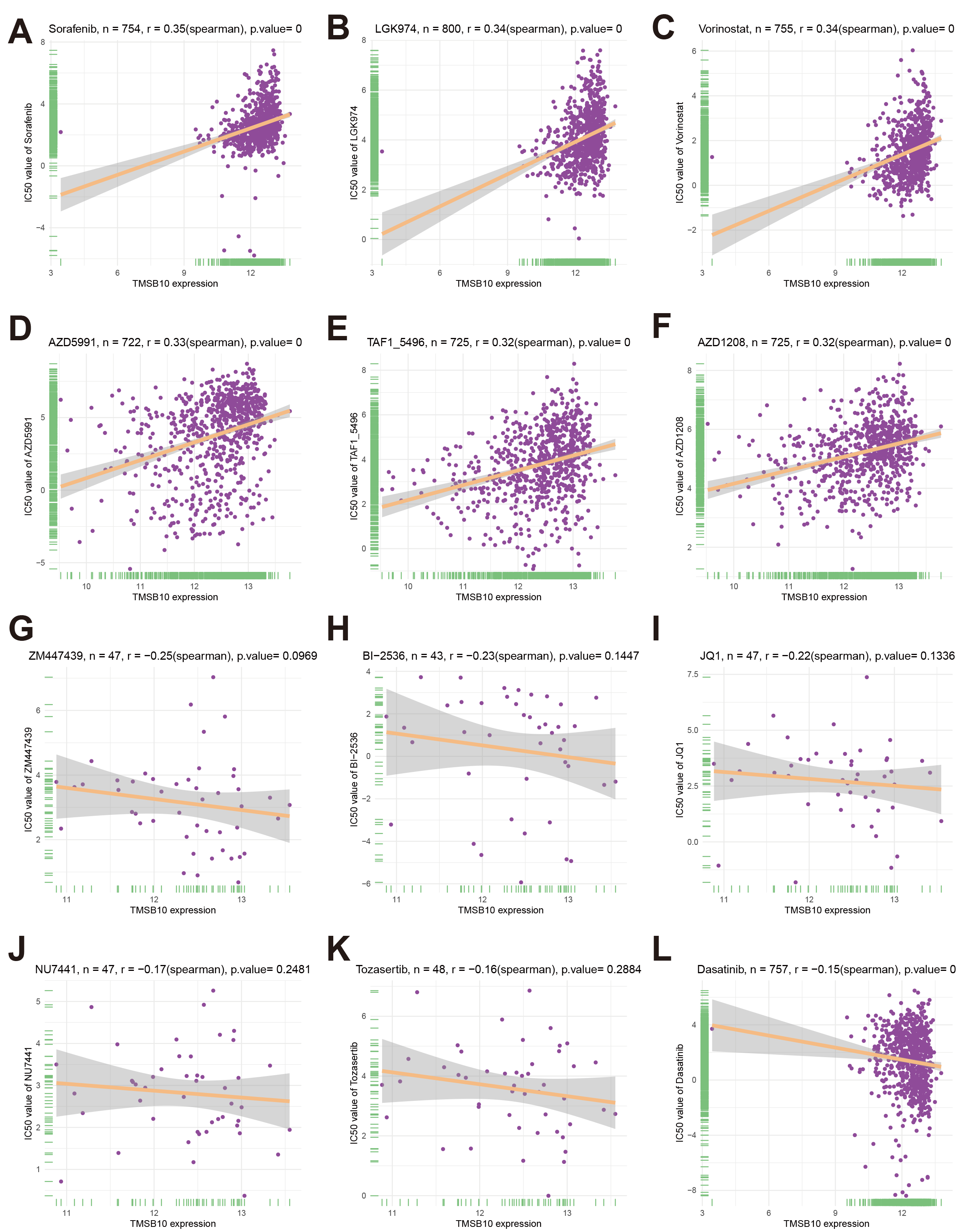

Supplement: Supplementary Figure 3 — Correlations of TMSB10 with Chemotherapy sensitivity. (A) Top 6 compounds showing resistant to TMSB10. (B) Top 6 compounds showing sensitivity to TMSB10. [file Image3.tif]

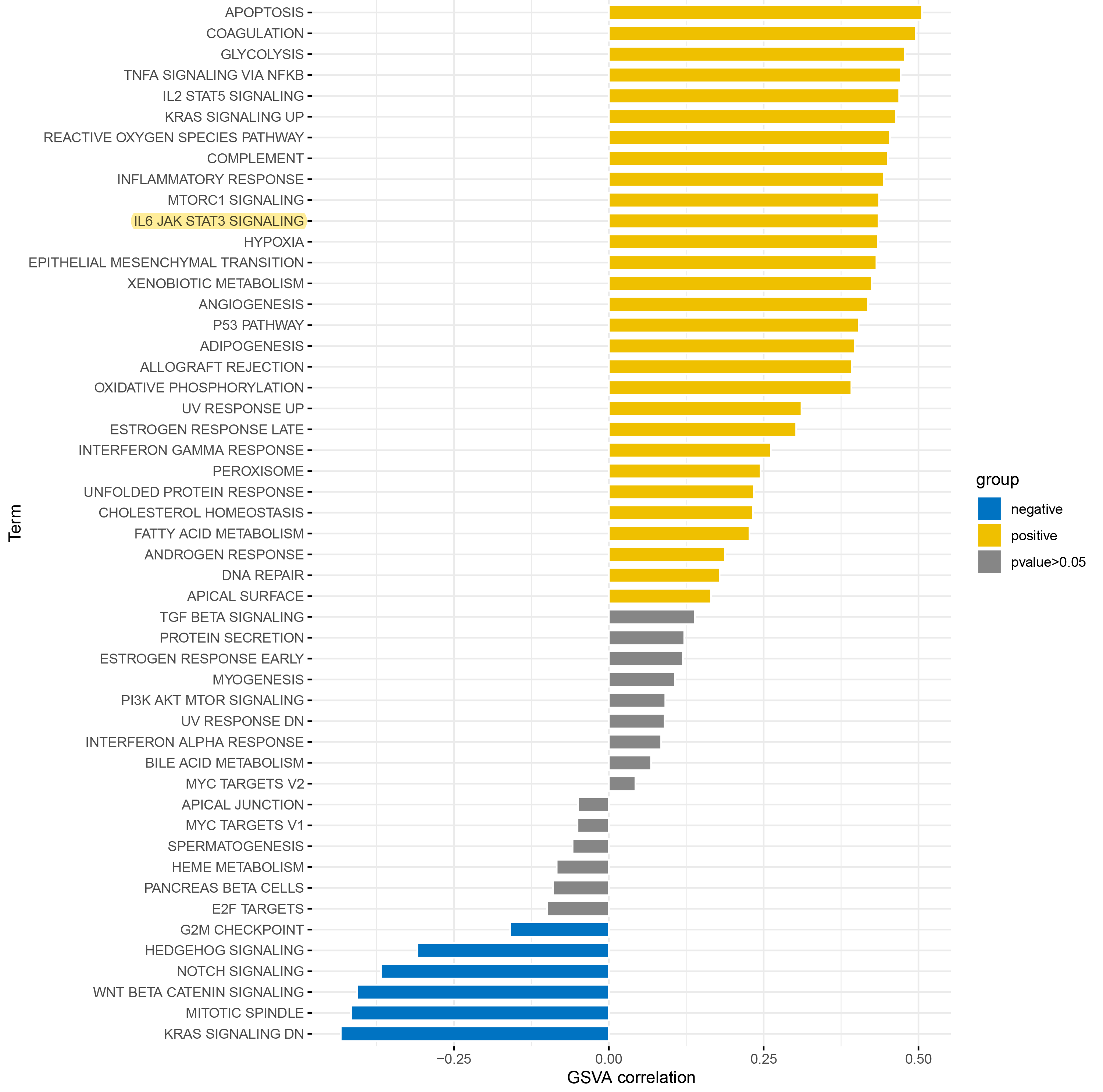

Supplement: Supplementary Figure 4 — Pathways enrichment analysis of TMSB10. [file Image4.tif]

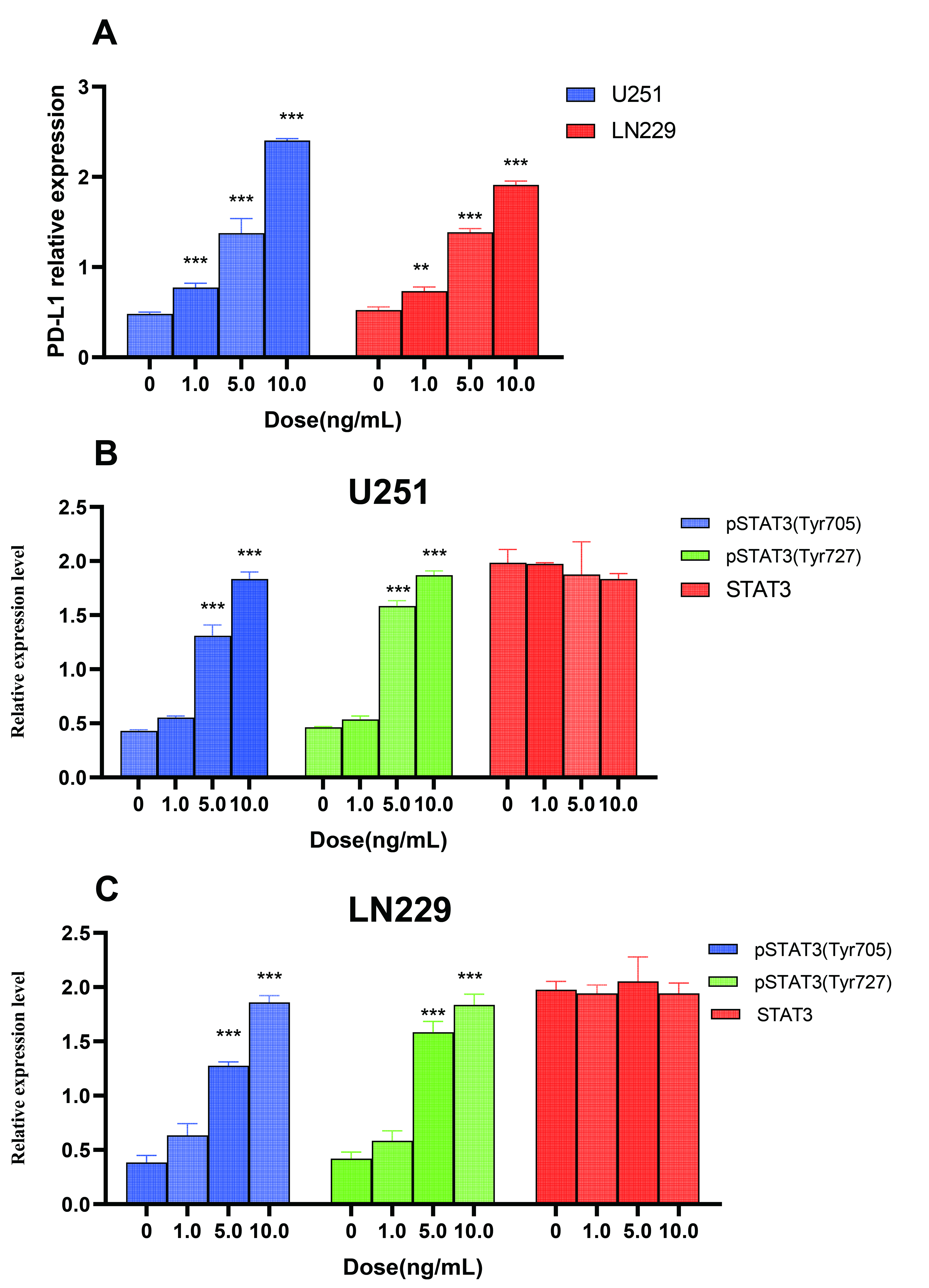

Supplement: Supplementary Figure 6 — IL-6 affect the PD-L1 (A) and p-STAT3 levels (B, C). [file Image6.tif]
